# DESIGNER FAT CELLS: ADIPOGENIC DIFFERENTIATION OF CRISPR-CAS9 GENOME-ENGINEERED INDUCED PLURIPOTENT STEM CELLS

**DOI:** 10.22203/eCM.v046a09

**Published:** 2023-12-29

**Authors:** E.V. Ely, A.T. Kapinski, S.G. Paradi, R. Tang, F. Guilak, K.H. Collins

**Affiliations:** 1 Department of Orthopedic Surgery, Washington University in Saint Louis, Saint Louis, MO 63110, USA; 2 Shriners Hospitals for Children–St. Louis, St. Louis, MO 63110, USA; 3 Department of Biomedical Engineering, Washington University in Saint Louis, Saint Louis, MO 63130, USA; 4 Center of Regenerative Medicine, Washington University in Saint Louis, Saint Louis, MO 63110, USA; 5 Department of Orthopaedic Surgery, University of California, San Francisco, CA 94143, USA

**Keywords:** Obesity, adipocyte differentiation, adipose tissue, functional adipocytes, adipokine secretion, cell-based therapies, tissue engineering

## Abstract

Adipose tissue is an active endocrine organ that can signal bidirectionally to many tissues and organ systems in the body. With obesity, adipose tissue can serve as a source of low-level inflammation that contributes to various co-morbidities and damage to downstream effector tissues. The ability to synthesize genetically engineered adipose tissue could have critical applications in studying adipokine signaling and the use of adipose tissue for novel therapeutic strategies. This study aimed to develop a method for non-viral adipogenic differentiation of genome-edited murine induced pluripotent stem cells (iPSCs) and to test the ability of such cells to engraft in mice *in vivo*. Designer adipocytes were created from iPSCs, which can be readily genetically engineered using CRISPR-Cas9 to knock out or insert individual genes of interest. As a model system for adipocyte-based drug delivery, an existing iPSC cell line that transcribes interleukin 1 receptor antagonist under the endogenous macrophage chemoattractant protein-1 promoter was tested for adipogenic capabilities under these same differentiation conditions. To understand the role of various adipocyte subtypes and their impact on health and disease, an efficient method was devised for inducing browning and whitening of Ipsc-derived adipocytes in culture. Finally, to study the downstream effects of designer adipocytes *in vivo*, we transplanted the designer adipocytes into fat-free lipodystrophic mice as a model system for studying adipose signaling in different models of disease or repair. This novel translational tissue engineering and regenerative medicine platform provides an innovative approach to studying the role of adipose interorgan communication in various conditions.

## Introduction

Obesity is a rapidly increasing public health condition affecting about one-third of the world population and is characterized by metabolic disturbance and pathological adipose signaling (Hildreth *et al*., 2021). Although adipose tissue was long considered to be inert, the active endocrine roles of adipocytes, a major cell type in adipose tissue, and other immune cell populations resident in adipose tissue have pleiotropic roles in both maintaining health as well as contributing to disease and reduced healthspan (Kahn *et al*., 2019; Kajimura, 2017). For example, low-level systemic inflammation from obesity results in metabolic dysfunction, inducing a milieu of obesity-linked diseases (Scarpellini and Tack, 2012) like diabetes mellitus (Singh *et al*., 2013), cardiovascular disease (Singh *et al*., 2013), and various musculoskeletal disorders (Anandacoomarasamy *et al*., 2008; Collins *et al*., 2018; Griffin and Guilak, 2008; King *et al*., 2013; Velasquez and Katz, 2010). These interactions have been difficult to disentangle due to interorgan crosstalk (Kirk *et al*., 2020; Velez *et al*., 2023) between adipose and downstream effector tissues (Kirk *et al*., 2020; Qi *et al*., 2023) but could be the key to understanding the onset and progression of diseases at the interface of aging and obesity or in multi-organ aging.

Adipose tissue exists in three phenotypically and functionally distinct subtypes – white adipose tissue (WAT), brown adipose tissue (BAT), and beige adipose tissue (BeAT) — which are distributed heterogeneously throughout the body in anatomical regions called depots (Marcadenti and de Abreu-Silva, 2015). Adipokines mediate inflammation and regulate systemic metabolic, skeletal, and reproductive processes (Griffin *et al*., 2009; Kwon and Pessin, 2013), but when dysregulated, adipokines can directly influence many obesity-associated diseases (Fasshauer and Blüher, 2015; Unamuno *et al*., 2018). Some examples of adipokines include adiponectin, leptin, visfatin, resistin, interleukin 6 (IL-6), and tumor necrosis factor alpha (TNF-α) (Kwon and Pessin, 2013). Conversely, BAT is considered a potential therapeutic (Tharp *et al*., 2015; Tharp and Stahl, 2015) and metabolically healthy tissue associated with improved caloric expenditure and thermal regulation (Marcadenti and de Abreu-Silva, 2015; Nicholls and Locke, 1984). An improved understanding of the physiologic and pathologic roles of adipose tissue will provide new insights toward controlling and leveraging adipose signaling for novel disease therapeutics in various disease states (Batún-Garrido *et al*., 2018; Grewal and Buechler, 2023; Recinella *et al*., 2020; Su *et al*., 2019) (i.e., COVID-19, cardiovascular disease, diabetes, cancer, metabolic syndrome). It may be similarly possible to harness adipose tissue phenotypes – such as BAT in the studies mentioned above – for enhanced therapeutic effects. However, the mechanisms by which specific adipose tissue subtypes and their unique signaling attributes affect disease progression are unknown, primarily due to a lack of well-controlled adipose tissue models. As such, the focus of this work is to develop a well-controlled model of engineered adipose tissue to better understand these mechanisms (Dani *et al*., 2022).

Numerous animal models have been utilized to study the effects of obesity, largely involving single gene knockout or transgenic mice and/or obesogenic diets to study metabolic changes (Coleman, 1978; Collins *et al*., 2015; Griffin *et al*., 2010; Griffin and Guilak, 2008; Griffin *et al*., 2009; Hariri and Thibault, 2010; Levin and Dunn-Meynell, 2002). More recently, fat-free lipodystrophic (LD) mice, coupled with implanted fat pads or mouse embryonic fibroblasts (MEFs) have been used to study the effects of different aspects of adipose signaling on musculoskeletal health (Collins *et al*., 2022; Collins *et al*., 2021). However, the heterogeneity of MEFs can result in the restoration of circulating adipokines that are known to contribute to proinflammatory catabolic activities and metabolic complications (Collins *et al*., 2021; Ferguson *et al*., 2018) (i.e., IL-6). Due to the inability to tightly control MEF signaling, developing induced pluripotent stem cells (iPSCs) as a cell source was a logical step to precisely engineer adipocytes.

iPSCs have multiple advantages as a source for developing “designer” cells, as single clonal iPSCs can be genome-edited, expanded, and differentiated widely into a variety of cell and tissue types (Diekman *et al*., 2012; Zomer *et al*., 2015). iPSCs have been successfully differentiated into adipocytes by overexpressing *Pparg* using adenovirus vector-mediated transient transduction (Mohsen-Kanson *et al*., 2014; Tashiro *et al*., 2009) and pharmacological overexpression using rosiglitazone or troglitazone (Fayyad *et al*., 2019; Kim *et al*., 2014; Tchoukalova *et al*., 2000). Developing a streamlined, virus-free protocol for adipogenesis allows users to generate large numbers of designer adipocytes from single clonal iPSCs consistently without concerns of losing differentiation potential. This reproducible method can be easily scaled up for clinical translation. This proposed model of designer adipocytes can be implanted into animal models of adipose ablation (Collins *et al*., 2021; Huang-Doran *et al*., 2010; Klaus *et al*., 1998; Savage, 2009), like lipodystrophic mice, to directly disentangle the role of adipokine signaling in downstream signaling, homeostasis, and disease progression. The overall goal of this study was to use iPSCs to genome-engineer designer adipose tissue to serve as a regenerative medicine platform to unravel the roles of adipokines in disease progression. Four phenotypes of designer adipocytes were generated, spanning mechanistic and drug delivery applications.

## Methods

### PDiPSCs isolation and culture

Murine induced pluripotent stem cells (iPSCs) were isolated and cultured from tail fibroblasts of adult C57BL/6 mice that were reprogrammed through forced overexpression of *Oct4, Sox2*, *Klf4*, and *c-Myc* as reported previously (Diekman *et al*., 2012). In the current study, four different lines of iPSCs were used: unedited/wildtype (WT), leptin knockout (*Lep*^−/−^, described below), Ccl2-Luciferase (Ccl2-Luc), and Ccl2-IL1Ra. Ccl2-Luc and Ccl2-IL-1Ra iPSCs were previously created in the lab by incorporating luciferase or IL-1Ra under the endogenous *Ccl2* promoter (Brunger *et al*., 2017a; Brunger *et al*., 2017b; Pferdehirt *et al*., 2022a). iPSCs were then differentiated into pre-differentiated iPSCs (PDiPSCs) through a high-density micromass culture, as previously described (Diekman *et al*., 2012), which directs them towards a mesenchymal state. From this procedure, PDiPSCs were attained and either frozen at passage 1 or maintained through adipogenic or expansion media culture.

### Monolayer adipogenesis culture

To create a streamlined protocol for PDiPSC-adipogenic differentiation, two methods were compared: (1) overexpression of *Ppargγ* using lentivirus and (2) the use of commercially available adipogenic media for MSCs. The virus used in this experiment was pMSCV-Pparγ virus, which was previously constructed and described (Tontonoz *et al*., 1994). For these comparisons, PDiPSCs were first plated on gelatin coated 6-well plates (150239; Thermo Fisher, Waltham, MA, USA) or 8-well cell culture slides (CCS-8; MatTek, Ashland, MA, USA) for 1 day at a density of 4.16 × 10^4^ cells/cm^2^ (4.16 × 10^5^ cells/well) in expansion media consisting of high glucose Dulbecco’s modified Eagle’s medium (11-995-081; Gibco, Waltham, MA, USA), fetal bovine serum (S11550; Atlanta Biologicals lot #A17004, Flowery Branch, GA, USA), 2-mercaptoethanol (21-985-023; Fisher Scientific, Hampton, NH, USA), ITS+ premix (AR014; R&D Systems, Minneapolis, MN, USA), penicillin-streptomycin (P4333–100ML; Sigma-Aldrich, St. Louis, MO, USA), l-ascorbic acid 2-phosphate (50 μg/mL; A8960–5G; Sigma-Aldrich), L-proline (40 μg/mL; P5607–25G; Sigma-Aldrich), and transforming growth factor–β3 (10 ng/mL; 243-B3–200; R&D Systems). On day 1 of culture, cells were transduced with equal titers of virus as previously described (Tontonoz *et al*., 1994), where titers were described as doses. Cells received either a low dose (3.1 μL/cm^2^), a high dose (5.2 μL/cm^2^), or no virus in 2.5 mL polybrene-containing expansion media without ascorbate and proline. On day 2, all wells were washed 2 times with growth media. On day 3, wells were puromycin-selected with 2.5 μg/mL of puromycin. On day 4, wells received either expansion media commercially available adipogenic media (Mesencult Adipogenic Differentiation Kit; 05505; STEMCELL, Vancouver, Canada). Media was changed and cells were collected for analysis every other day up to day 14. The standard culture conditions referred to in the subsequent methods are PDiPSCs cultured in expansion or adipogenic media, with no virus.

### Generation of leptin knockout iPSCs

Leptin knockout (KO) iPSCs were created utilizing resources from the Genome Engineering & iPSC Center at Washington University in St. Louis. Using CRISPR-Cas9, two guide RNAs (gRNAs) (YH665.m.Lep.sp7 and YH666.m.Lep.sp9) were selected based on an off-target analysis and distance to the target site as seen below (Table 1). Murine leptin has 2 exons and thus a deletion approach was used to complete the knockout.

### Leptin supplement for adipogenesis culture

In culture, the leptin knockout PDiPSCs received doses of leptin to test whether loss of adipogenesis can be rescued under standard culture conditions. Leptin knockout PDiPSCs in culture were supplemented with 1,000 ng/mL of murine leptin (498-OB-05M; R&D Systems, Minneapolis, MN, USA), a concentration that was determined after performing a leptin dose analysis. The literature supports doses anywhere from 250–1,000 ng/mL (Picó *et al*., 2022; Wagoner *et al*., 2006). Cells received this dosage with each media change from the time they were plated until the end of the adipogenesis time course.

### Brown and white adipocyte culture

Various adipogenic media were assessed for their ability to efficiently generate distinct adipocyte sub-populations from iPSCs in monolayer. WT PDiPSCs were plated at a density of 4.16 × 10^4^ cells/cm^2^. Cells were incubated for 24 hours in 5 % CO2 at 37 °C, then fed with iPSC expansion media (described above) thereafter until 80–90 % confluency was reached (1–2 days). Upon exhibiting the desired confluency, PDiPSCs were cultured in the presence of the different fat-specific media types. Cells were maintained in either a PDiPSC expansion media control or one of the following commercially available adipogenesis media: STEMCELL Mesencult MSC Basal Medium with adipogenic supplement (Mesencult Adipogenic Differentiation Kit; 05505; STEMCELL, Vancouver, Canada) (adipogenic media), AdipoQual BAT Medium (OS-013; Obatala Sciences, New Orleans, LA, USA) (brown media), or Obatala AdipoQual WAT Medium (OS-014; Obatala Sciences, New Orleans, LA, USA) (white media). Cells were cultured in their respective media for up to 7 days of adipogenesis, with media changes taking place every other day. Sample collections occurred at days 0, 1, 3, 5, and 7 for staining and imaging.

### Mouse embryonic fibroblast culture

Mouse embryonic fibroblasts (MEFs) were used as a control cell line in this study as previous work demonstrated great success with implanting these cells into a mouse model (Collins *et al*., 2021; Ferguson *et al*., 2018). MEFs were prepared as previously described (Ferguson *et al*., 2018) and cultured at a density of 4.16 × 10^4^ cells/cm^2^ in expansion or adipogenic media for up to 14 days.

### Gene expression

Samples for gene expression analysis were harvested and total RNA was isolated from the cell according to recommendations of the manufacturer (48300; Norgen Biotek, Thorold, ON, Canada). Reverse transcription was performed using the superscript VILO complementary DNA synthesis kit (11755500; Life Technologies, Carlsbad, CA, USA) following the manufacturer’s instructions. Quantitative polymerase chain reaction (qPCR) was performed using FASTSybr (4385617; Applied Biosystems, Waltham, MA, USA) following manufacturer’s instructions. Cycling parameters were initial denaturation at 95 °C for 10 minutes followed by 40 cycles of 95 °C for 15 seconds and 60 °C for annealing and extension for 60 seconds. Gene fold changes were determined relative to a control group using 18S ribosomal RNA as a reference gene. Data are reported as fold changes and were calculated using the 2^−ΔΔCt^ method. Primer pairs were synthesized by Integrated DNA Technologies, Inc.

### Inflammatory challenge

To measure the kinetics of IL-1Ra production in the Ccl2-IL1Ra PDiPSCs, WT and Ccl2-IL-1Ra PDiPSCs were treated with expansion and adipogenesis media until day 14. On day 14, the cells were challenged with 1 ng/mL murine IL-1α (400ML005CF; Fisher Scientific). 500 μL of culture media was collected and snap frozen at 0, 4, 12, and 72 hours post inflammatory challenge. Control groups were not challenged with IL-1α.

### Enzyme-linked immunosorbent assays

Enzyme-linked immunosorbent assay (ELISA) was used to measure the concentration of IL-1ra *in vitro* using a mouse IL-1Ra DuoSet ELISA kit (DY480; R&D Systems) with a 1:10 dilution. Each sample was measured in technical duplicates and absorbance was measured at 450 and 540 nm (n = 4–6 per group). The lower limit of detection for the ELISA was 156 pg/mL.

### Histological characterization

#### Oil Red O staining

Oil Red O is a stain for neutral lipids and is used to assess adipocyte differentiation. All lines of PDiPSCs were characterized qualitatively by Oil Red O staining to assess morphology and lipid content. A stock solution was created by adding 0.5 g Oil Red O (O0625–25G; Sigma-Aldrich) to 100 mL isopropanol. A working solution was curated from the stock solution using 3 parts stock solution and 2 parts deionized (DI) water. Cells were fixed with 4 % paraformaldehyde for 15 minutes. Paraformaldehyde was then aspirated and then 1 mL of working solution was added to the each well of a 6-well plate to cover the cells for 30 minutes. Working solution was then aspirated and the wells were washed 1× with DI water. DI water was added back into the wells and cells were imaged using a Cytation 5 (Biotek; Agilent, Santa Clara, CA, USA).

#### BODIPY stain

PDiPSCs cultured in 8-well cell culture slides (CCS-8; Mattek) were stained with BODIPY 493/503 (4,4-Difluoro-1,3,5,7,8-Pentamethyl-4-Bora-3a,4a-Diaza-*s*-Indacene) (D3922; Invitrogen, Waltham, MA, USA), a green, fluorescent dye that stains lipids, and a DAPI (4′,6-Diamidino-2-Phenylindole, Dilactate) (422801; BioLegend, San Diego, CA, USA) counterstain. Briefly, cells were fixed with 4 % paraformaldehyde for 5 minutes and then washed 2× with PBS. A 1:200 BODIPY solution was added to each well for 20 minutes. The wells were then washed 3× with PBS. A 1:1,000 DAPI solution was added to each well for 7.5 minutes. The wells were then washed 3× with PBS and then the slide was cover slipped using ProLong Glass Antifade Mountant (P36982; Invitrogen). Slides were then imaged using confocal microscopy (LSM 880, Zeiss, Thornwood, NY, USA).

### Image analysis

#### Quantifying differentiation efficiency

A custom ImageJ workflow was developed to quantify adipocyte differentiation efficiency from PDiPSCs and MEFs during adipogenesis. First, the total number of adipocytes were manually counted from 20× BODIPY/DAPI images. Cells were deemed adipocytes if they showed positive BODIPY staining surrounding DAPI-labeled nuclei. For nuclei surrounded by low signal, the corresponding cells were considered adipocytes if adjacent regions contained distinct, circular lipid droplets, and not hazy, irregularly shaped, or low signal. To obtain an overall cell count, the DAPI image channel was first pre-processed for nuclei segmentation. Pre-processing steps included subtracting a value of 50 pixels across the entire image background. A low-pass filter was then applied to blur the image (Gaussian blur) with a sigma value of 4. An ImageJ plugin (StarDist (Schmidt *et al*., 2018)) was then utilized to measure the total number of nuclei in the resulting image. Finally, the differentiation efficiency was calculated by dividing the number of differentiated adipocytes by the total number of cells in an image (n = 3 images/condition).

#### Quantifying lipid droplet size

A separate ImageJ workflow was developed for the quantification of lipid droplet content and size among PDiPSCs and MEF cells under various conditions undergoing adipogenesis. Briefly, 20× confocal images containing only the BOPIDY channel were first pre-processed for lipid droplet segmentation by converting to image type 16-bit and subtracting up to a 50-pixel radius across the entire image background to correct for hazy signaling due to cellular autofluorescence. The background-subtracted image was duplicated, after which a low-pass filter was applied to blur the duplicate image (Gaussian blur, σ = 4) and smooth intensity variations across the image. The blurred image was subtracted from the non-blurred image. The result of this operation was converted to a 32-bit image type for manual threshold application. A manual threshold value of 3 was applied to yield a binary image of segmented lipid droplets. To quantify lipid droplet size for the depot-specific adipogenesis experiments, a particle analysis was performed across the full image, yielding individual particle areas. The average of these area measurements was found for the image. This process was repeated for at least three images of the same condition.

#### Quantifying lipid droplet content

To quantify lipid droplet content, 5 cells were randomly selected from the binary image for further analysis. Target cells were selected by first utilizing the corresponding DAPI-only image as a reference to choose a single nucleus in one of five image quadrants (top left/right, bottom left/right, center). Then, the corresponding cell of interest was located in the binary image by referencing common target cell features between both the DAPI-only image and the multi-channel image. Upon locating target cell droplets in the binary image, the total areas of these particles were measured by tracing around the lipid-containing region of interest and enacting the particle analysis function. The perimeter of the target cell was then manually traced in the multi-channel image and total cell area was measured. For each of the 5 selected cells, the total lipid content was calculated by dividing the sum of the particle areas by the total area of the cell. The average lipid content was then determined across the 5 cells of the same image. This process was repeated for at least three images of the same condition.

### *In vivo* cell delivery

To perform observations of cell delivery over time, we used fat free lipodystrophic (LD) mice (adiponectin-DTA) (Wu *et al*., 2018) characterized by a low-level systemic inflammatory environment that can drive the Ccl2-Luciferase circuit for *in vivo* imaging (Brunger *et al*., 2017b; Collins *et al*., 2021). First, to understand the concentration of cells to inject into the mice, Ccl2-Luc PDiPSCs were cultured until day 3 in adipogenic media then removed from tissue culture plastic using trypsin for 10 minutes to help remove excess matrix. Then, cells were centrifuged for 3 minutes at 1500 rpm (500–600 g) and counted. Cells were resuspended to yield final concentrations of either 10 or 20 million cells in 250 μL sterile PBS for injection. Control LD mice received injections of 250 μL PBS (n = 3). LD mice were anesthetized using 2–3 % isoflurane. Using a 27-gauge insulin syringe, cells were injected just superficial to the sternum slowly until the contents of the injection were delivered to the mice. Once the ideal cell number was determined (20 million cells per injection), we performed a 28-week injection experiment using LD and WT mice. All animal protocols were approved by the Washington University School of Medicine IACUC under protocol 19–0774.

### *In vivo* imaging

Mice were prepared for imaging using Veet hair removal and standard clippers. For the cell number experiments, LD mice were imaged weekly for 4 weeks. Mice were imaged under 2–3 % isoflurane anesthetic. D-luciferin substrate (150 mg/kg in phosphate-buffered saline (PBS); Gold Biotechnology, St. Louis, MO, USA) was delivered by the Molecular Imaging Core via intraperitoneal injection (IP). After waiting 10 minutes, mice were positioned on their dorsal aspect and images were taken from the ventral view with an exposure time of 5 minutes using an IVIS Lumina (PerkinElmer, Waltham, MA, USA; Living Image 4.2; 1-min exposure; bin, 8; field of view, 12.5 cm; f/stop, 1; open filter). Images were analyzed by the Molecular Imaging Core and a pre-defined region of interest (ROI) 1 cm^2^ area superficial to the mouse sternum, operationalized as photon flux. Once the cell number experiment was optimized, a new subset of mice was imaged at 1, 2, 6, 9, 12, and 28 weeks timepoints.

### Statistics

Statistical analysis and sample numbers for each experiment are detailed in the respective figure legends. All statistics were performed in Graphpad Prism 8 (Graphpad Software, San Diego, CA, USA). Data are presented as means ± standard error of the mean.

## Results

### PDiPSCs demonstrate adipocyte phenotype after virus-free culture

**Fig. 1.**
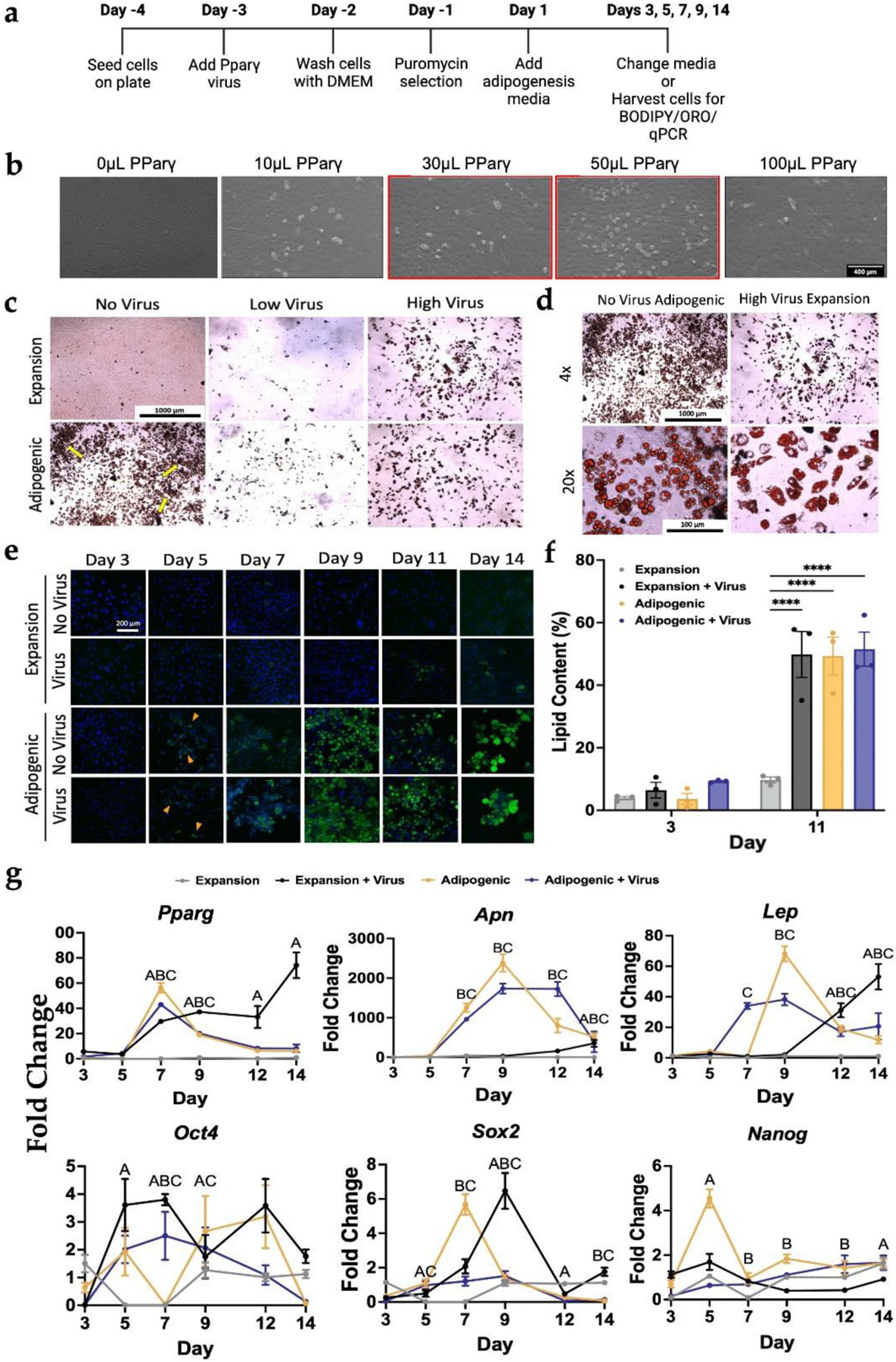
PDiPSC adipogenesis using either viral and virus-free methods. (**a**) Timeline of culture conditions. (**b**) WT PDiPSCs were cultured with varying concentrations of *Pparγ* virus. After 14-day culture in expansion media with virus, 50 μL of Pparγ virus displays the highest concentration of adipocyte-like cells. 100 μL of virus resulted in less cell density. Scale bar is 400 μm. All groups received 5 μL Puromycin. WT PDiPSCs were then cultured with no *Pparγ* virus, a low dose of *Pparγ* virus (3.125 μL/cm^2^), or high dose of *Pparγ virus* (5.208 μL/cm^2^) in expansion or adipogenic media for 14 days. (**c**) All groups were stained with Oil Red O and imaged at 4×. All groups were stained with Oil Red O stain, where the red stain indicates lipid content in cells. Scale bar is 1000 μm. (**d**) 20× images of virus-free adipogenic media and virus-expansion media groups were taken to examine morphology of the PDiPSCs. Scale bars are 1000 μm and 100 μm. (**e**) WT PDiPSCs with no virus and virus were cultured in expansion and adipogenic media conditions for 14 days and stained with BODIPY/DAPI. BODIPY, green, stains for lipids and DAPI, blue, stains nuclei. Scale bar is 200 μm. (**f**) Lipid content of the PDiPSCs was measured at days 3 and 11 using the BODIPY images. Bars represent lipid content (%) ± SEM (*n = 3*). *Asterisks* represent significance (**** *p* < 0.00005) compared with the WT PDiPSCs expansion media control. (**g**) Cells from all groups above were collected at various timepoints for gene expression characterization. *Pparg, Apn*, and *Lep*, *Oct4, Sox2*, and *Nanog* were target genes evaluated. were target genes evaluated. Values represent fold change ± SEM (*n = 3*). Letters represent significance (A *p* < 0.05, expansion media + virus; B *p* < 0.05 adipogenic media; C *p* < 0.05 adipogenic media + virus). All statistics were run using a 2-way ANOVA with Sidak’s post-hoc test. SEM, standard error of the mean.

To minimize the perturbations to the cells, a virus-free protocol was developed for PDiPSCs by based on a commercially available adipogenic media designed for MSC adipogenesis. We then compared this protocol to published methods using lentiviral transduction of *Ppar*γ, a transcription factor integral for the differentiation of mesenchymal-like cells to adipocytes (Fig. 1a). Increasing concentrations of the virus were tested, and 50 μL of the virus, or the high virus dose [5.208 μL/cm^2^], displays the highest concentration of rounded, lipid-like cells, while 30 μL of the virus, or the low dose [3.125 μL/cm^2^], yields the second-highest concentration of lipid-like cells (Fig. 1b). Oil Red O (ORO) staining of samples at day 14 with different (no, low, and high) virus concentrations and media demonstrated differentiation of lipid-containing cells using expansion media for both the low and high doses of virus, adipogenic media with both low and high doses of virus, and adipogenic media with no virus (Fig. 1c). For the virus conditions, the highest virus concentration [5.208 μL/cm^2^] yielded the highest density of PDiPSCs stained with ORO in both expansion and adipogenic media conditions. In contrast, the low virus condition resulted in fewer lipid-containing cells overall. As such, the highest concentration virus dose was used as a control to confirm PDiPSC-adipocyte differentiation in a virus-free manner. Under 20× magnification, the adipogenic media group with no virus, expansion media group with virus, and the adipogenic media group with no virus revealed cells with a rounded, plump morphology in clusters of 3–4 cells, and ringlets of lipids were plentiful. The expansion group with virus also demonstrated a rounded morphology, but fewer clusters were observed compared to the adipogenic media group (Fig. 1d).

A time course experiment was used to evaluate PDiPSC adipogenesis with and without the virus. Cells were fixed 3-, 5-, 7-, 9-, 11-, and 14-days after plating and stained with BODIPY/DAPI (Fig. 1e). In the expansion media groups, few cells stained positive from days 3–11. In the expansion media condition with the virus, some BODIPY stain was evident on day 14. In the adipogenic media groups, BODIPY staining was present as early as day 5 in the virus and no-virus groups. The density of BODIPY-stained PDiPSCs illustrated a trend toward increased staining in both adipogenic media groups with time up to day 14.

Image analysis tools were used to quantify prior observations with the BODIPY time course images to quantify lipid content per cell in each image (Fig. 1f). Lipid content was quantified as the percentage of lipid content per cell. For this, PDiPSCs cultured in expansion or adipogenic media with virus or no virus were compared on day 3 and day 11. Results of these analyses revealed no significant differences between all treatment groups from the control expansion media condition at day 3 (expansion with virus 6.4 ± 2.5 %, adipogenic no virus 3.6 ± 1.8 %, adipogenic with virus 9.3 ± 0.2 %). However, by day 11, all groups demonstrated significantly increased (*p* < 0.001) percent lipid content than the control (expansion with virus 49.9 ± 7.4 %, adipogenic no virus 49.4 ± 6.0 %, adipogenic with virus 51.5 ± 5.5 %). Together, these data indicated no overt benefit to using virus compared to adipogenic media to differentiate PDiPSCs into adipocytes based on morphological presentation.

### PDiPSCs demonstrate increased expression of adipogenic genes in virus free culture

RT-qPCR studies were performed to explore differences in mRNA expression in virus and no virus expansion media and adipogenic media conditions over a 14-day time course. Transcriptional changes during adipogenesis were measured by evaluating pluripotency and adipogenic marker gene expression in cultured PDiPSCs.

*Pparg* mRNA levels were significantly increased in all groups on day 7 and 9 compared to control expansion media (*p* < 0.001) (Fig. 1g). At day 11 and 14, only the expansion media with virus group had significantly increased expression when compared to mRNA levels measured in the control group (*p* < 0.001). Strikingly, peak *Pparg* expression for the adipogenic media condition was at day 7 (56.4 ± 3.9 fold change), suggesting that PDiPSCs differentiated into adipocytes by the later time points. We next evaluated adiponectin, *Apn*, which is highly expressed by mature adipocytes (Farmer, 2005), indicating commitment to adipogenesis. Adipogenic media groups with virus and no virus exhibited significantly higher mRNA levels of *Apn* starting at day 7 (*p* < 0.001), with peak fold changes observed at day 9 for both (no virus 2384.0 ± 221.6 fold change, with virus 1737 ± 125.9 fold change). These outcomes were consistent with the peak expression of *Pparg* at day 7, which would precede commitment to adipocytes. Moreover, the expansion media with virus group had significantly increased *Apn* expression compared to expansion media control only at day 14 *(p* < 0.001), suggesting that adipocyte differentiation occurred over varying time courses with different methods. Lastly, mRNA levels for leptin, *Lep*, whose circulating expression is proportional to the amount of fat in the body or the number of adipocytes present (Krempler *et al*., 1998; Perakakis *et al*., 2021), were evaluated. The adipogenic media group with virus exhibited significantly higher mRNA levels of *Lep* compared to the expansion media control by day 7 (*p* < 0.001), while the adipogenic group with no virus was significantly different from control and had peak mRNA expression at day 9 (68.2 ± 5.0 fold change, *p* < 0.001). The expansion media with virus condition peak *Lep* mRNA levels were observed at day 14 (*p* < 0.001). These findings were consistent with the trends observed in the other markers for adipogenesis. Taken together, mRNA fold changes for all three genes, *Pparg, Apn*, and *Lep* (Fig. 1g) were significantly increased using a virus-free approach compared to expansion media control groups as early as 5 days in culture. mRNA levels of key pluripotency markers octamer binding transcription factor 4 (*Oct4*), SRY-box transcription factor *(Sox2*), and nanog homeobox (*Nanog*) confirmed that the PDiPSCs committed to an adipogenic cell lineage and did not de-differentiate back into iPSCs from their mesenchymal-like PDiPSC state (Fig. 1g).

### CRISPR-Cas9-edited leptin knockout adipocytes

**Fig. 2.**
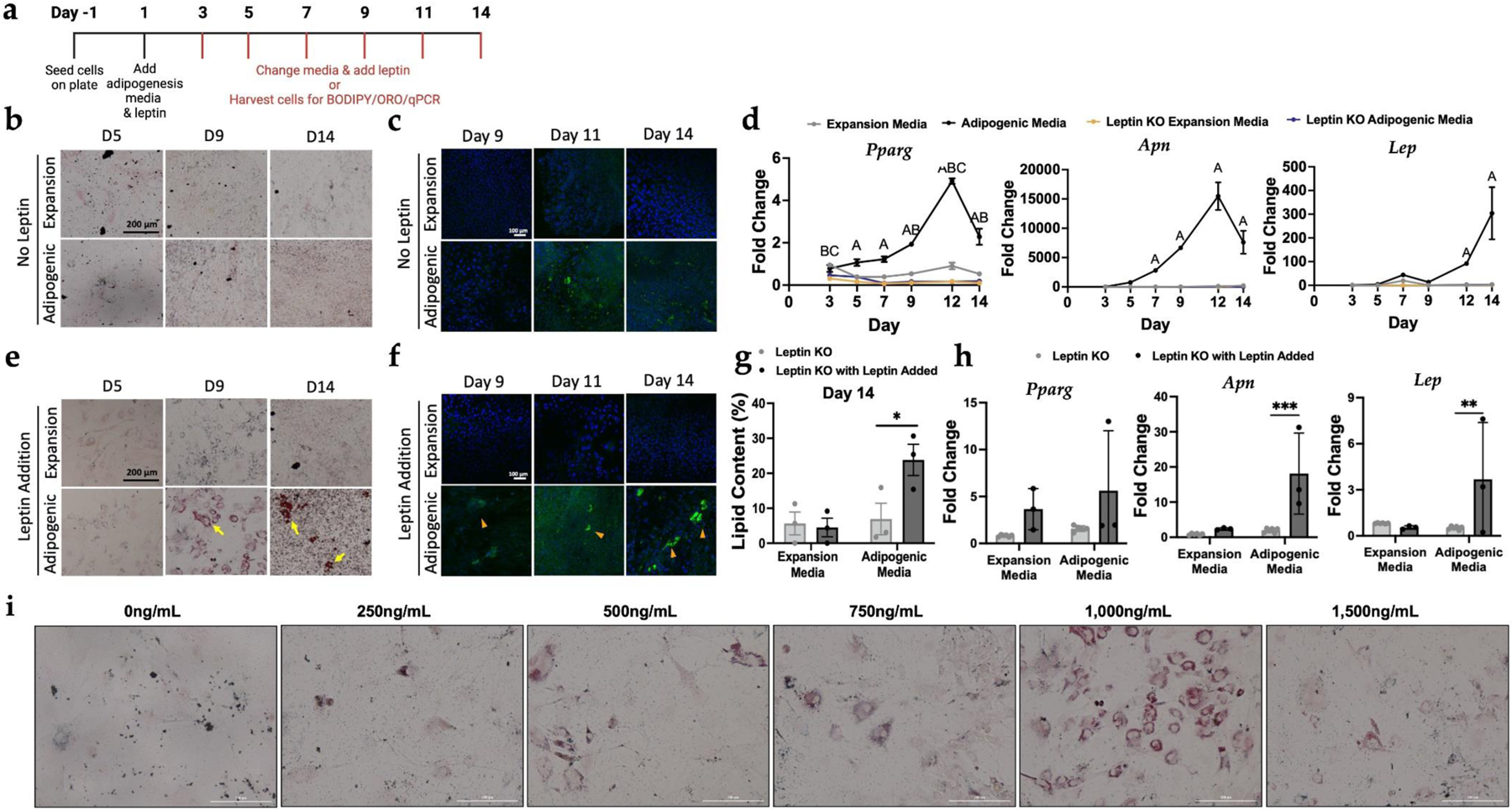
Leptin knockout PDiPSCs are capable of adipocyte differentiation, but not without supplemental leptin. Leptin KO PDiPSCs were cultured in expansion or adipogenic media for 14 days. (**a**) Timeline of culture conditions. (**b**) All groups were stained with Oil Red O and Days 5, 9, and 14 are shown here. Red stain indicates lipid content in cells. The yellow arrows point to lipid-containing cells. Scale bar is 200 μm. (**c**) All Groups were stained with BODIPY/DAPI BODIPY, green, stains for lipids and DAPI, blue, stains nuclei. Scale bar is 100 μm. (**d**) WT and leptin KO PDiPSCs were collected at various timepoints for gene expression characterization. *Pparg, Adip*, and *Lep*, were target genes evaluated. Values represent fold change ± SEM (*n = 3*). Letters represent significance (A *p* < 0.05, WT adipogenic media; B *p* < 0.05 leptin KO expansion media; C *p* < 0.05 leptin KO adipogenic media) from WT PDiPSCs expansion media control. In an effort to restore adipogenic differentiation, murine leptin was added to the culture media as a supplement. (**e**) Leptin KO PDiPSCs with leptin added were cultured in expansion or adipogenic media and for 14 days and were stained with Oil Red O and days 5, 9, and 14 as shown here. Scale bar is 200 μm. (**f**) The same groups as (**e**) were stained with BODIPY/DAPI. Scale bar is 100 μm. (**g**) Lipid content of the PDiPSCs (leptin KO and leptin KO with leptin added) was measured at day 14 using the BODIPY images. Bars represent lipid content (%) ± SEM (*n = 3*). *Asterisks* represent significance (* *p* < 0.01) compared with the leptin KO PDiPSCs in adipogenic media. (**h**) Cells from leptin KO with and without leptin added were collected at various timepoints for gene expression characterization, day 9 is shown here. *Pparg, Apn*, and *Lep*, were target genes evaluated. Values represent fold change ± SEM (*n = 3*). *Asterisks* represent significance (** *p* < 0.0001, *** p < 0.00001) compared with the leptin KO PDiPSCs expansion media control. (**i**) For 9 days, Leptin KO PDiPSCs were treated with adipogenic media and supplemented with varying doses of leptin. Samples were fixed and stained with Oil Red O. 1,000 ng/mL of leptin added to culture media displays the highest concentration of lipid-containing cells. Scale bar is 200 μm. All statistics were run using a 2-way ANOVA with Sidak’s post-hoc test. SEM, standard error of the mean.

To establish a platform for modifying adipokine genes with CRISPR-Cas9 and systematically testing the impact of secreted factors from engineered designer adipocytes, we initially employed engineered leptin knockout (KO) adipocytes as a proof of concept. We hypothesized that PDiPSCs with a leptin knockout would display robust adipogenesis following the validated virus-free culture protocol (Fig. 2a). Leptin KO cells grown in expansion and adipogenic media showed no positive staining with ORO, indicating no measurable presence of lipids. Days 5, 9, and 14 are shown as references (Fig. 2b). To confirm this finding, BODIPY fluorescent staining for lipids was conducted, which similarly did not reveal any minor positive staining until day 11 and day 14 in the adipogenic media group (Fig. 2c). Consistent with previous reports of leptin’s role in cell differentiation (Scheller *et al*., 2010), leptin KO cells proliferated rapidly and at times, were overconfluent, and lifted off the bottom of the culture well using the typical cell seeding conditions. To better understand the lack of adipocyte-like morphology, mRNA levels of key adipogenic genes were measured (Fig. 2d). WT PDiPSCs in adipogenic media had significantly higher (*p* < 0.001) gene expression compared to the expansion media control, while surprisingly, the two leptin KO conditions had significantly lower (*p* < 0.001) *Pparg* mRNA levels compared to control. WT PDiPSCs in adipogenic media had significantly higher mRNA levels (*p* < 0.001) of *Apn* compared to the control, while the leptin KO PDiPSCs were similar to control. mRNA levels of *Lep* were not detected in leptin KO PDiPSCs. Together, these data suggest leptin is required for PDiPSC adipogenic differentiation, and as such, this hypothesis was directly tested next.

### Leptin supplementation in culture media rescues adipogenesis in leptin knockout iPSCs

To restore the typical growth kinetics of PDiPSCs with adipogenesis media, leptin was added to the cell culture media. Using the same measures of morphology and gene expression as above, restoration of adipogenesis was observed in leptin KO PDiPSCs with leptin added. At days 9 and 14, leptin KO PDiPSCs with leptin added in adipogenic media revealed positive lipid staining by ORO (Fig. 2e). Similarly, BODIPY staining revealed adipocytes in culture, demonstrated by green stain and surrounding blue DAPI stained nuclei (Fig. 2f). Using the BODIPY images, percent of lipid content per cell was quantified for the leptin KO groups with and without leptin added (Fig. 2g). Leptin KO PDiPSCs with leptin added in adipogenic media had a significantly higher lipid content compared to leptin KO PDiPSCs with no leptin added (no leptin 6.9 ± 4.5 %, leptin added 23.8 ± 4.5 %, *p* = 0.028). qPCR profiles were measured at day 9 and the leptin KO with leptin added group was compared to leptin KO samples for *Pparg, Apn*, and *Lep* gene expression (Fig. 2h). *Pparg* mRNA levels were not significantly increased when leptin was added to the culture media (expansion media *p* = 0.286, adipogenic media *p* = 0.112). However, *Apn* and *Lep* mRNA levels were significantly increased in the leptin KO adipogenic condition with leptin added (*Apn p* < 0.001, *Lep p* = 0.007). Taken together with the morphological outcomes in this cell line, adding leptin back into the system rescued adipogenesis. Varying leptin concentrations were tested to determine the optimal dose to recover adipogenesis in the leptin KO PDiPSCs, and 1,000 ng/mL results in the highest density of lipid-laden cells, as demonstrated by ORO (Fig. 2i).

### Designer adipocytes as an anti-cytokine therapy to mitigate inflammation

**Fig. 3.**
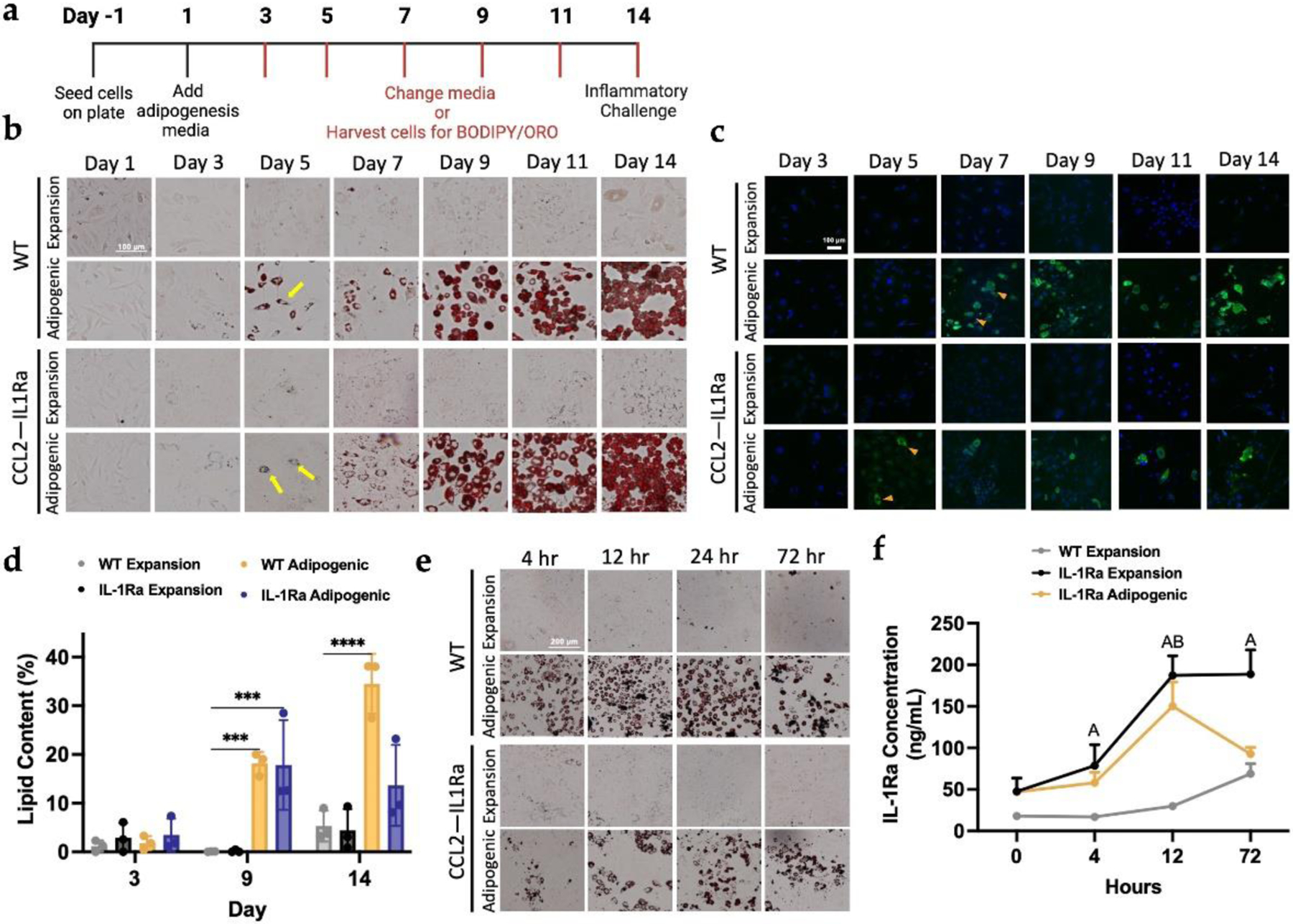
Adipogenic differentiation of genome-edited self-regulating anti-inflammatory iPSCs. WT PDiPSCs and Ccl2-IL1Ra PDiPSCs were cultured in expansion or adipogenic media for 14 days and samples were fixed every other day from day 1 to day 14. (**a**) Timeline of culture conditions. (**b**) All groups were stained with Oil Red O stain, where the red stain indicates lipid content in cells. The yellow arrows point to lipid-containing cells. Scale bar is 200 μm. (**c**) WT and Ccl2-IL1Ra PDiPSCs were stained with BODIPY/DAPI at various timepoints. BODIPY, green, stains for lipids and DAPI, blue, stains nuclei. Scale bar is 100 μm. (**d**) Lipid content of the PDiPSCs was measured at days 3, 9, and 14 using the BODIPY images. Bars represent lipid content (%) ± SEM (*n = 3*). *Asterisks* represent significance (*** *p* < 0.0005, **** *p* < 0.00005) compared with the WT PDiPSCs expansion media control. At Day 14, WT and Ccl2-IL1Ra PDiPSCs were dosed with an inflammatory challenge to test the self-regulatory circuit. (**e**) WT and PDiPSCs were fixed 4-, 12-, 24-, and 72-hours after inflammatory challenge and stained with Oil Red O. (**f**) An ELISA was performed to determine the production of IL1-Ra protein using media from various timepoints of the inflammatory challenge. Values represent mean ± SEM (*n = 3*). Letters represent significance (A *p* < 0.05, IL1Ra+expansion media; B *p* < 0.05 IL1Ra+adipogenic media). All statistics were run using a 2-way ANOVA with Sidak’s post-hoc test. ELISA, enzyme-linked immunosorbent assay; SEM, standard error of the mean.

Using self-regulating iPSCs developed in a previous study (Brunger *et al*., 2017a; Brunger *et al*., 2017b; Choi *et al*., 2021; Pferdehirt, 2019; Pferdehirt *et al*., 2022a), we developed engineered adipocytes capable of producing interleukin-1 receptor antagonist (IL-1Ra) in the presence of low-level inflammation. We tested the ability of Ccl2-IL1Ra PDiPSCs (IL-1Ra) to differentiate into adipocytes using the previously validated protocol and to maintain the ability to produce IL-1Ra post-differentiation (Fig 3a). IL-1Ra PDiPSCs had similar morphology compared to WT PDiPSCs in culture, as shown in the ORO and BODIPY figures (Fig. 3b,c). In the ORO images, IL-1Ra and WT PDiPSCs demonstrated rounded lipid droplets at day 5. The number of lipid-containing cells increased with time in culture, indicated by the red staining (Fig. 3b). Between days 9–14, peak adipogenesis was observed by morphological and mRNA readouts for both groups. The BODIPY with DAPI counterstain illustrated that the IL-1Ra PDiPSCs in adipogenic media differentiated slightly earlier than the WT PDiPSCs at day 5, but both groups in adipogenic media displayed consistent adipogenesis through day 14 (Fig. 3c). Lipid content was quantified at days 3,9, and 14, and WT and IL1Ra groups in expansion and adipogenic media were compared to the control WT expansion media group (Fig. 3d). At day 3, all groups had similar mean percentage of lipid content (WT adipogenic 1.80 ± 0.84 %, IL-1Ra expansion 2.9 ± 1.8 %, IL-1Ra adipogenic 3.5 ± 1.9 %). By day 9, there were significant differences in the WT and IL-1Ra adipogenic media groups. The WT PDiPSCs in adipogenic media mean lipid content was 18.2 ± 1.4 % while the IL-1Ra PDiPCSs in adipogenic media had a mean lipid content of 17.9 ± 5.3 % with a wide range of values from 12.5–28.5 %. These data suggest that the IL-1Ra PDiPSCs may differentiate into adipocytes less efficiently under these conditions but can differentiate along the 14-day time course.

Ccl2-IL1Ra PDiPSCs were then analyzed to determine if the designer adipocytes produced IL-1Ra to similar levels of cells in expansion media in the presence of low-level inflammation. WT and IL-1Ra PDiPSCs were challenged with IL-1α and assessed for morphological and protein production changes. At the various timepoints, WT and IL-1Ra PDiPSCs stained positively for ORO, indicating that the cells persisted in culture throughout the inflammatory treatment (Fig. 3e). Protein production of IL-1Ra was quantified for each group along the 72-hour time course and compared to the control WT PDiPSCs in expansion media at each timepoint. Twelve hours after the inflammatory challenge, the IL-1Ra PDiPSCs produced a significantly higher amount of IL-1Ra (expansion media 188.0 ± 23.6 ng/mL, *p* < 0.001, adipogenic media 150.0 ± 28.9 ng/mL, *p* < 0.001) compared to the control (29.9 ± 2.5 ng/mL) (Fig. 3f). At 72 hours, the IL-1Ra PDiPSCs in expansion media again produced a significantly higher amount of IL-1Ra (188.0 ± 29.1 ng/mL, *p* < 0.001) compared to the control, but the IL-1Ra PDiPSCs in adipogenic media did not produce a significantly higher amount compared to the control (92.6 ± 7.9 ng/mL, *p* = 0.577). Therefore, rewired Ccl2-IL1Ra PDiPSCs designer adipocytes can produce anti-inflammatory mediators in the presence of an inflammatory environment.

### iPSC-derived brown and white adipocytes

**Fig. 4.**
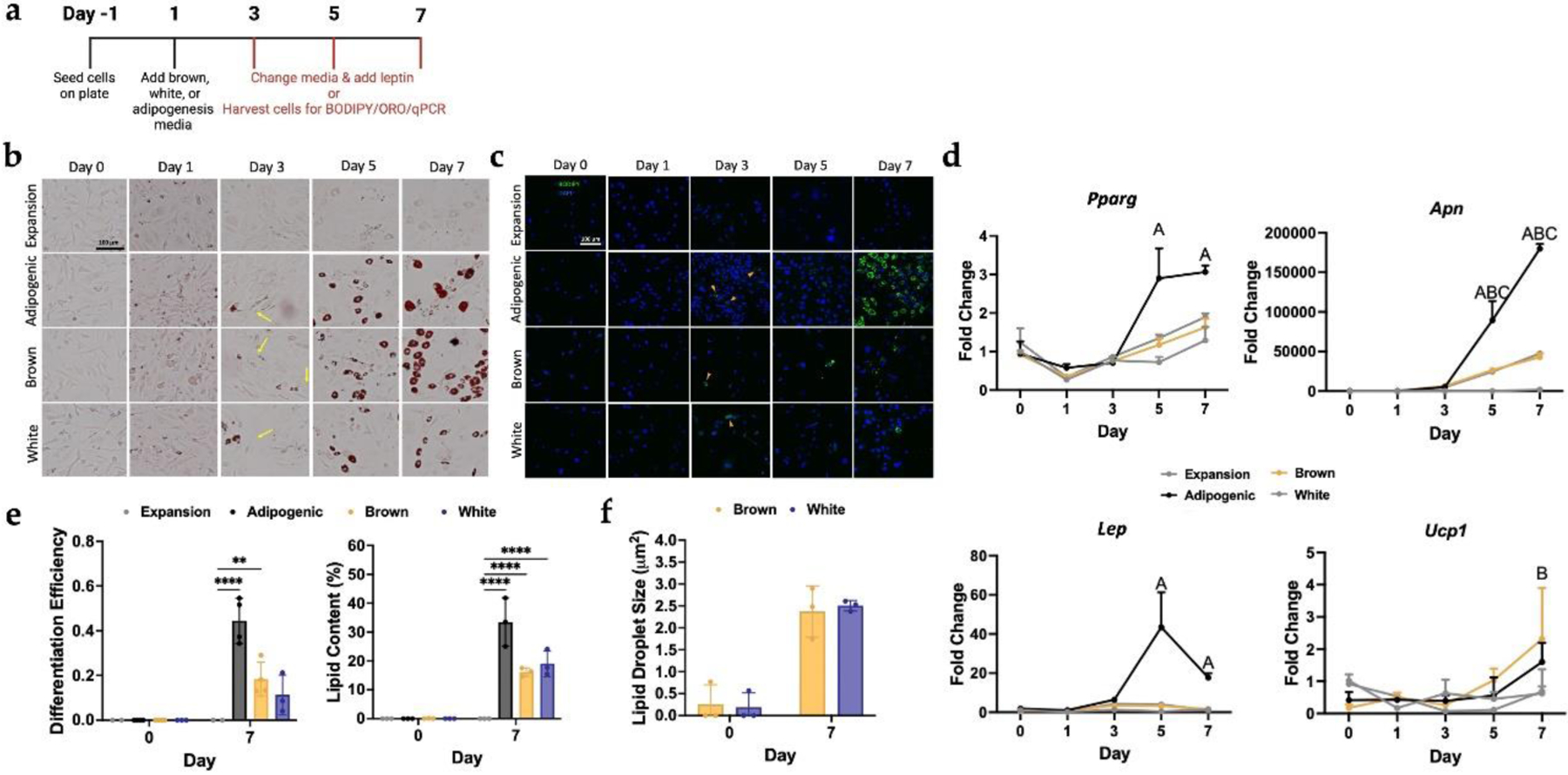
Brown and White PDiPSCs display adipocyte-like morphology. WT PDiPSCs were cultured with various media (expansion, adipogenic, brown, and white) for 7 days. (**a**) Culture conditions and timeline. (**b**) All groups were stained with Oil Red O stain, where the red stain indicates lipid content in cells. The yellow arrows point to lipid-containing cells. Scale bar is 100 μm. (**c**) All groups were stained with BODIPY/DAPI at various timepoints. BODIPY, green, stains for lipids and DAPI, blue, stains nuclei. Scale bar is 100 μm. (**d**) Cells from all groups were collected at various timepoints for gene expression characterization. *Pparg, Apn*, *Lep*, and *Ucp1* were target genes evaluated. Values represent fold change ± SEM (*n = 3*). Letters represent significance (*A p* < 0.05*, adipogenic media; B p* < 0.05 *brown media; C p* < 0.05 *white media*). (**e**) Differentiation efficiency and lipid content of all groups was measured at days 0 and 7 using the BODIPY images. Bars represent differentiation efficiency or lipid content (%) ± SEM (*n = 3*). *Asterisks* represent significance (** *p* < 0.005, **** *p* < 0.00005) compared with the WT PDiPSCs expansion media control. (**f**) Lipid droplet size was measured at days 0 and 7. Bars represent lipid droplet size ± SEM (*n = 3*). *Asterisks* represent significance (** *p* < 0.005, *** *p* < 0.0005, **** *p* < 0.00005) compared with the WT PDiPSCs expansion media control. All statistics were run using a 2-way ANOVA with Sidak’s post-hoc test. SEM, standard error of the mean.

We sought to determine if PDiPSCs fed AdipoQual BAT or WAT media for white and brown adipogenesis would be capable of differentiating into distinct populations of brown and white adipocytes in culture (Fig. 4a). ORO images taken from day 0 to day 7 illustrated that the brown and white PDiPSCs behaved similarly to WT PDiPSCs, where lipid droplets appeared in culture by day 3 (Fig. 4b). By day 7, the WT and Brown PDiPSCs qualitatively appeared to have the highest number of lipid-containing cells but were closely followed by the white PDiPSCs. These initial data demonstrated that the AdipoQual BAT and WAT media were capable of inducing adipogenesis. Next, samples were stained with BODIPY and DAPI to determine if the lipid containing cells observed in ORO staining were adipocytes (Fig. 4c). Initial signs of adipocytes were spotted on day 3, but unlike the Oil Red O, the BODIPY images indicated fewer lipid-containing cells. The brown PDiPSCs differentiated into more adipocytes than the white PDiPSCs, which was consistent with the ORO findings. Differentiation efficiency and percent lipid content were quantified using the BODIPY confocal images (Fig. 4e). WT PDiPSCs in adipogenic media, brown PDiPSCs, and white PDiPSCs were compared to control WT PDiPSCs in expansion media at day 0 and day 7. Of note, at day 7, the WT adipogenic (0.44 ± 0.05, *p* < 0.001) and brown PDiPSCs (0.18 ± 0.04, *p* < 0.007) had significantly higher differentiation efficiencies compared to the WT control. Although not statistically significant, there was a trend toward increased differentiation efficiency of the white PDiPCs (0.11 ± 0.05, *p* < 0.145) compared to the control.

Lipid sizes of the brown and white PDiPSCs were quantified to determine if brown PDiPSCs exhibited smaller lipid sizes than the white PDiPSCs. Lipid size at day 0 and day 7 was evaluated, and there were no significant changes in lipid size amongst any of the groups (Fig. 4f). Brown adipocytes at day 7 had a mean lipid droplet size of 2.4±0.33μm and white adipocytes had a mean lipid size of 2.50 ± 0.07 μm. We hypothesize that at later time points, we will see a more robust difference between the two groups, especially given the wide range of lipid sizes for brown PDiPSCs and tight sample range for white PDiPSCs.

Furthermore, selected adipogenic markers were analyzed to investigate whether the brown and white PDiPSCs displayed adipogenic phenotypes and, most importantly, whether the brown PDiPSCs were characterized by *Ucp1* expression, the defining marker for brown adipose (Fig. 4d). Only the WT adipogenic group had significant changes in gene expression for *Pparg (p* < 0.001) when compared to the WT expansion media control. The brown and white PDiPSC groups were not significantly different from the control group from day 0 to day 7. All groups had significantly increased *Apn* expression compared to the control (*p* < 0.001) by days 5 and 7. *Lep* mRNA levels were significantly increased in the WT adipogenic group compared to control (*p* < 0.021) on day 7. mRNA levels of *Ucp1* were increased at day 7 in the brown PDiPSC group compared to the control (*p* < 0.048). These data suggest that, in addition to white adipocytes, brown UCP1 producing adipocytes can be differentiated from iPSCs.

### iPSC-derived adipocytes have similar morphology to MEFs *in vitro*

**Fig. 5.**
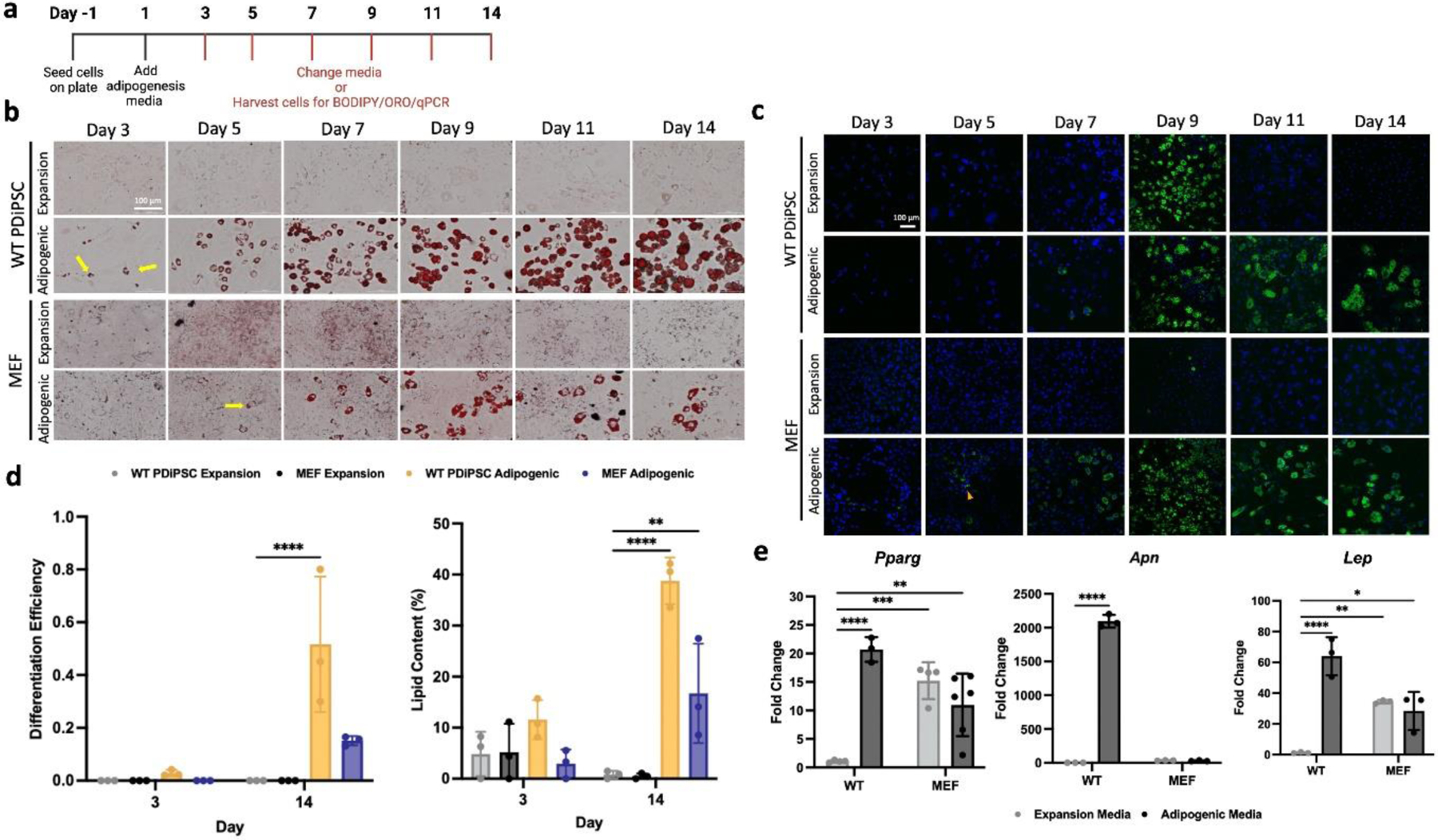
PDiPSCs and MEFs display similar adipogenic morphology in culture. WT PDiPSCs and MEFs were cultured in expansion or adipogenic media for 14 days. (**a**) Culture conditions timeline. (**b**) All groups were stained with Oil Red O stain, where the red stain indicates lipid content in cells. The yellow arrows point to lipid-containing cells. Scale bar is 100 μm. (**c**) All groups were stained with BODIPY/DAPI at various timepoints. BODIPY, green, stains for lipids and DAPI, blue, stains nuclei. Scale bar is 100 μm. (**d**) Differentiation efficiency and lipid content of all groups was measured at days 3 and 14 using the BODIPY images. Bars represent differentiation efficiency or lipid content (%) ± SEM (*n = 3*). *Asterisks* represent significance (* *p* < 0.05, ** *p* < 0.005, *** *p* < 0.0005, **** *p* < 0.00005) compared with the PDiPSCs expansion media control. (**e**) Cells from all groups above were collected at various timepoints for gene expression characterization, day 9 is shown here. *Pparg, Apn*, and *Lep* were target genes evaluated. Bars represent fold change ± SEM (*n = 3*). Letters represent significance (A *p* < 0.05, PDiPSCs adipogenic media; B *p* < 0.05 MEFs expansion media; C *p* < 0.05 MEFs adipogenic media). All statistics were run using a 2-way ANOVA with Sidak’s post-hoc test. SEM, standard error of the mean.

To evaluate if iPSCs and MEFs demonstrate similar phenotypes for adipogenesis *in vitro*, morphology was evaluated and compared between PDiPSCs and MEFs grown in monolayer with expansion and adipogenic media (Fig. 5a). The PDiPSCs in adipogenic media demonstrated evidence of lipid containing cells by day 3, as seen by the positive ORO staining (Fig. 5b), while the MEFs in adipogenic and expansion media conditions started to form lipid containing cells around day 5. As the number of PDiPSCs appeared to increase with time in adipogenic media, the same trend was not observed for the MEFs. The MEFs in adipogenic media generated similar numbers of lipid containing cells from days 7–14. Interestingly, the MEF expansion media condition did show some indication of lipid containing cells, although they appeared less phenotypically healthy and round compared to the adipogenic media condition. PDiPSCs and MEFs were stained with BODIPY to determine if the lipid containing cells were adipocytes (Fig. 5c). The BODIPY staining demonstrated adipogenesis in the MEF adipogenic media condition at around day 5, and the number of adipocytes appeared to increase throughout the 14-day time course. Similar trends were observed with the PDiPSC adipogenic media condition. Lipid content and differentiation efficiency were quantified from the BODIPY confocal images to understand if there were any differences between MEFs and PDiPSCs grown *in vitro* (Fig. 5d). With respect to the percent of lipid content at day 14, both the PDiPSC (38.7 ± 2.64 %, *p* < 0.001) and MEF (16.7 ± 6.62 %, *p* < 0.003) groups grown in adipogenic media had significantly increased percentages of lipid content compared to the PDiPSC expansion control group. The PDiPSC adipogenic media condition had a significantly increased differentiation efficiency (0.52 ± 0.15, *p* < 0.001) compared to the control group, while the MEF adipogenic group did not (0.15 ± 0.01, *p* < 0.166). MEFs and PDiPSCs were evaluated for adipogenic gene expression over time and were compared to PDiPSCs in expansion media as the control. mRNA levels for *Pparg* in both MEF groups grown in expansion and adipogenic media had significantly higher gene expression compared to the control (expansion media *p* < 0.001, adipogenic media *p* < 0.005) (Fig. 5e). However, these levels were reduced compared to the PDiPSC adipogenic media condition (*p* < 0.001). *Apn* expression was analyzed, and neither MEF group was significantly different from the control, unlike the PDiPSC adipogenic media condition (*p* < 0.001). Lastly, *Lep* expression was also different between the cell types. MEF groups demonstrated significantly higher mRNA levels for *Lep* (expansion media *p* < 0.005, adipogenic media *p* < 0.016), but these levels were still lower than the PDiPSC adipogenic group (*p* < 0.001).

### Designer adipocytes engraft and are functional *in vivo* for 28 weeks

**Fig. 6.**
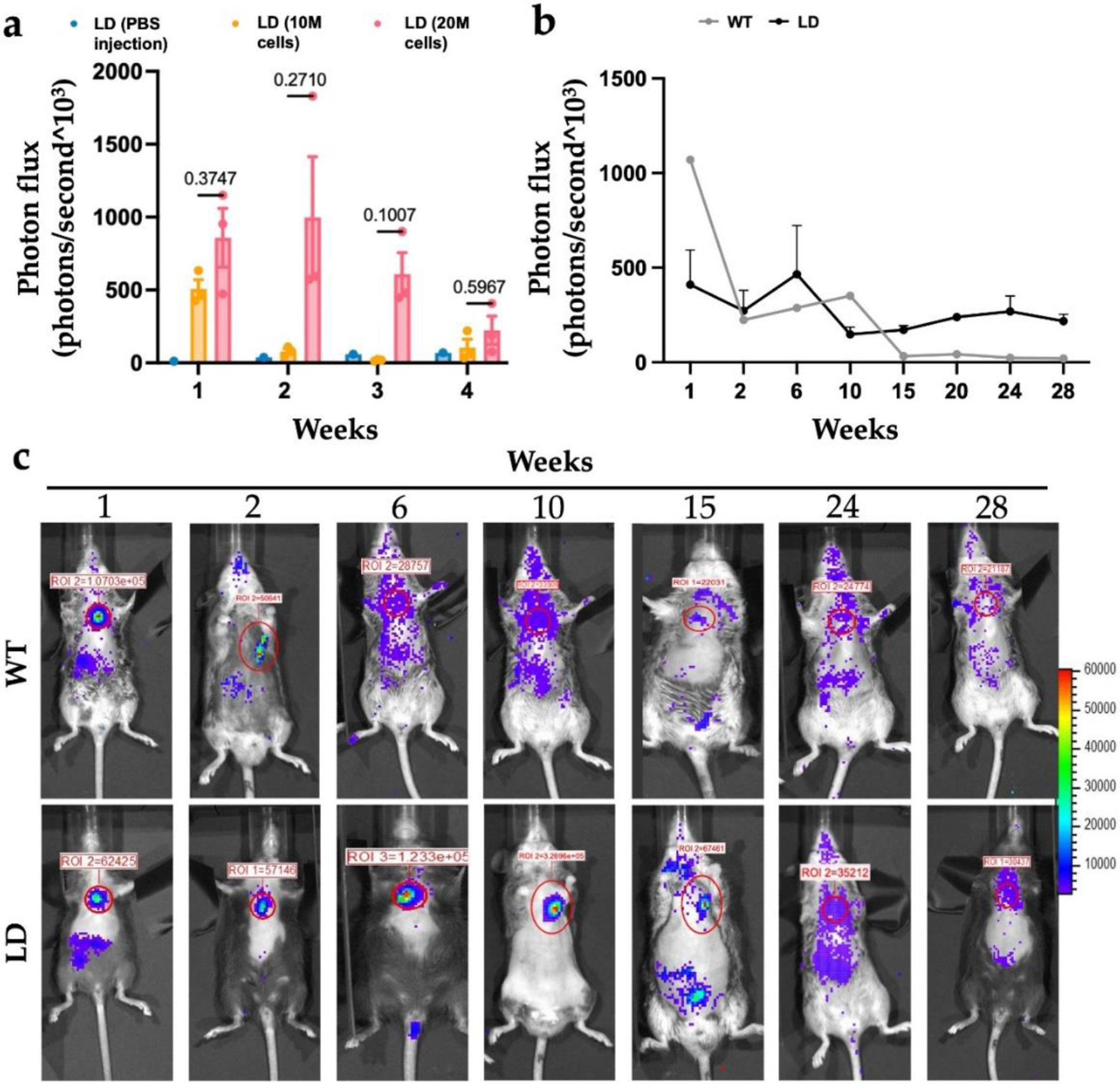
Self-regulating adipogenic iPSCs are viable and functional *in vivo* for up to 28 weeks. Different cell concentrations of Ccl2-Luciferase PDiPSCs primed in adipogenic media for 3 days were injected into LD mice and IVIS imaging was used to quantify cells present in the mouse. (**a**) Photon flux per animal was calculated every week for 4 weeks. Bars represent mean photon flux ± SEM (*n = 3–7*). Exact p values are displayed using LD mice with PBS injection as the control. (**b**) WT and LD mice were injected with 20 million cells and imaged for 28 weeks using IVIS imaging. Values represent mean photon flux ± SEM (*n = 3–7*). All statistics were run using a 2-way ANOVA with Sidak’s post-hoc test. SEM, standard error of the mean. (**c**) Representative images of WT and LD mice at various timepoints from IVIS imaging are shown. The red circle highlights the area of injection.

To image and measure cell signaling over time, we utilized Ccl2-Luciferase iPSCs (Ccl2-Luc), which were engineered to produce luciferase under the endogenous Ccl2-promoter in the presence of low-level inflammation (Brunger *et al*., 2017a; Brunger *et al*., 2017b; Hao and Baltimore, 2009). Mice were injected with a PBS control, 10 million, or 20 million adipocyte media primed Ccl2-Luciferase PDiPSCs and then imaged using IVIS imaging (Fig. 6a). Mice given the higher cell dose, 20 million cells, had a consistently higher photon flux compared to the mice given 10 million cells and were trending or statistically significant from control PBS injection at week 1 (*p* < 0.059) and week 2 (*p* < 0.031) across the 4 weeks after injection. A longer-term *in vivo* implantation study was then used to determine the length of time the implanted designer adipocytes were detectable *in vivo* (Fig. 6b,c). One week after injection, localized luminescent signal was present in the sternal region of WT and LD mice where the cells were injected (Fig. 6c). Two weeks after injection, the WT mice exhibited a marked decrease in luminescence in the sternal region, but the LD mouse maintained robust concentrated signaling. Then, six weeks after injection, there was a considerable reduction in localized luminescence in the WT mouse, and robust signaling was maintained in the LD mouse. However, after 10 weeks, localized luminescence was lost in both groups. These findings were corroborated by calculating the photon flux of both groups along the time course (Fig. 6b). After ten weeks, WT mice photon flux levels did not exceed that of background luminescence, while LD luminescence was reduced but detectable through 28 weeks.

## Discussion

In this study, designer adipocytes were created that can both individually dissect the role of adipokines in a variety of contexts and harness these cells to deliver anti-cytokine therapies. Importantly, this work demonstrated that adipocytes can be derived from murine iPSCs in a virus-free manner. CRISPR-Cas9 genome editing was then utilized to generate designer adipocytes without leptin, illustrating that leptin is required for iPSC-adipogenesis. The addition of leptin to culture media rescued the loss of adipogenesis and allowed for the growth of leptin KO adipose (Scheller *et al*., 2010). We then created a self-regulating adipocyte drug delivery system and confirmed that these iPSCs (Brunger *et al*., 2017a; Brunger *et al*., 2017b) can differentiate into adipocytes and produce similar drug levels compared to other differentiation protocols. We also derived white (Qi *et al*., 2023) and brown adipocytes (Hammel and Bellas, 2020) using a straightforward approach for iPSC differentiation. Lastly, we demonstrated that the designer adipocytes can be implanted into fat-free mice for a functional *in vivo* readout of cytokine signaling from adipose tissue.

Developing a non-viral protocol for iPSC adipogenesis was an essential focus of this work to minimize the number of modifications or perturbations with which the cells would be challenged. Given the goal of using CRISPR-Cas9 gene editing, which involves guide RNAs that may be delivered using viral vectors to knock out adipokines of interest, reducing the number of transfections the cells would experience was critical. Our data suggest that the virus-free adipogenesis protocol recapitulated morphology, lipid content, and adipogenic gene expression of well-known differentiation methods, and thus, we determined that iPSCs do not require a viral overexpression of *Ppar*γ to achieve robust adipogenesis. However, there is evidence that using virus to facilitate differentiation of human iPSCs is beneficial and may depend on the cell source (Qi *et al*., 2023; Qi *et al*., 2022). This simplified protocol facilitates the use of iPSCs as a renewable source for adipocytes.

Designer adipocytes can be derived from iPSCs with a single gene knockout and used to disentangle specific adipokines’ roles in various disease states directly. We validated this tool by first knocking out leptin in iPSCs, which once differentiated into adipocytes, can be used to directly unravel the role of leptin signaling in disease progression *in vivo*, independent of body weight and metabolism. While culturing the leptin knockout PDiPSCs using the validated adipogenesis protocol, we confirmed that leptin is an important regulator and inductor of adipogenesis *in vitro*, consistent with previous reports (Palhinha *et al*., 2019; Tencerova *et al*., 2019; Yue *et al*., 2016), and in this case, was required for PDiPSC adipogenesis. We observed that the leptin KO PDiPSCs grew in an uncontrolled manner and, at times, were overconfluent and lifted from the well when using typical cell seeding conditions. To rescue adipogenesis, we added murine leptin at physiological levels similar to those previously reported (Picó *et al*., 2022; Wagoner *et al*., 2006) to the cell culture media and were able to recover morphology, increase gene expression profiles, and importantly, we created a unique platform to individually test the mechanism of deleting various adipokines using genome engineering. Coupled with immuno-compromised or fat-free mice, these designer adipocytes enable researchers to directly test the mechanistic role of leptin-adipose signaling *in vivo* in a well-controlled manner.

To generate an adipocyte-based drug delivery strategy using iPSCs, we validated that Ccl2-IL-1Ra PDiPSCs, previously created by Brunger et al. (Brunger *et al*., 2017a; Brunger *et al*., 2017b), were capable of adipogenesis. Previously, these cells were differentiated into a chondrocyte-like phenotype to generate living anti-cytokine implants (Choi *et al*., 2021; Collins *et al*., 2023). The Ccl2-IL1Ra-derived adipocytes had similar morphological characterization and differentiation efficiencies and, importantly, maintained the ability to produce IL-1Ra to similar levels as those differentiated with expansion media in the presence of low-level inflammation. Of interest, we previously found that using a cell’s intrinsic promotor to transcribe and ultimately deliver IL-1Ra resulted in similar levels to constitutive delivery yet yielded better disease mitigation (Pferdehirt *et al*., 2022a). This finding not only illustrated intriguing therapeutic potential for cells, but also suggested that developing cell-based drug delivery strategies of known mediators and to accommodate new mediators as they are discovered (Choi *et al*., 2021). Overall, these findings outlined a new cell-based anti-cytokine therapeutic approach that can be used to combat the low-level inflammatory environment propagated by obesity (, and several other conditions.

A simplified approach to generating brown and white iPSC-derived adipocytes was explored in this study. We demonstrated that different media formulations (Obatala Sciences) are sufficient to induce brown or white adipogenesis using PDiPSCs from the morphological characterization and differentiation efficiency analysis. While we expected to see significant differences in lipid droplet size between the white and brown PDiPSC-derived adipocytes, as these are defining characteristics that distinguish the two, there was not a substantial difference in lipid droplet size between the two in these studies. This lack of a difference in lipid droplet size could be due to the timepoint we analyzed and is the focus of ongoing work. Significant changes in adipogenic gene expression were only observed for *Apn*, while *Pparg and Lep* were not significant by day 7 in culture. Importantly, *Ucp1*, a marker for brown adipocytes, was increased compared to the control. Perhaps at an early timepoint of 7 days, PDiPSCs can likely be successfully differentiated into brown and white adipocytes, but the culture must be analyzed through day 14 to see apparent phenotypic differences in the outcomes we assessed. Previous groups have successfully generated brown-like ADMSCs using genome engineering techniques and the beneficial effects of transplantation of these cells into obese mice, including improved glucose tolerance, insulin sensitivity, and increased energy expenditure (Qi *et al*., 2022; Tharp and Stahl, 2015; Wang *et al*., 2020; Zhang *et al*., 2021). This efficient approach of generating diverse types of adipose tissue from iPSCs is viable, and it will allow future applications to integrate genome editing with these tissues, creating new possibilities for cell-based therapeutics, as previously explored with adipose-derived stem cells (Lopez-Yus *et al*., 2023). By simplifying the downstream differentiation of these three phenotypes of adipocytes, we can then use genome engineering to knock out genes of interest or rewire the different adipocyte depots for drug delivery applications.

To enable translational use of these designer adipocytes, we demonstrated that these cells can engraft and survive after implantation into fat-free mice *in vivo* for up to 28 weeks. For these studies, we aimed to recapitulate the methods used to inject mouse embryonic fibroblasts subcutaneously into mice, as these proved successful in creating a fat depot 21 days after injection (Brenot *et al*., 2020; Collins *et al*., 2021; Ferguson *et al*., 2018). We utilized Ccl2-Luciferase PDiPSCs (Brunger *et al*., 2017a; Brunger *et al*., 2017b) primed with adipogenic media in culture for three days before injection into mice. We then tested the feasibility of using these implanted cells in a longer-term 28-week study. Detectable luciferase signal from the Ccl2-Luciferase cells was observed over the 28 weeks, concordant with our lab’s previous data (Collins *et al*., 2023); however, after six weeks, localized luminescence was lost at the sternal region where the cells were injected. These data highlight the feasibility of implanting these designer adipocytes *in vivo*, however, more work must be done to enhance this approach and cell survival in the mouse and characterize the potential role of biomaterials and connective tissue to create more robust implants (Anvari and Bellas, 2021; Di Caprio and Bellas, 2020). Our future work will further develop animal models to test these engineered cells.

## Limitations

While this study represents several advancements in the potential use of iPSCs to engineer adipocytes, there are also several limitations of these studies. First, when characterizing and evaluating the feasibility of creating distinct depots of adipocytes, there was a limited supply of media, so the 14-day time course used in other sections was abbreviated. In the abbreviated culture in this study, brown and white adipocytes were successfully differentiated using the media; however, the lack of significant findings in the gene expression characterization are likely due to the shortened culture timeline. Additionally, transplanting the designer adipocytes into mice highlights the need for a refined approach in future studies to increase localized cell engraftment, which is essential in various transplantation applications. The methodology from MEF transplantation was used and compared using PDiPSCs. There are differences that may point to why the MEFs are able to engraft into the host and why the PDiPSCs may disperse. MEFs are a heterogeneous “pre-differentiated” population where PDiPSCs have undergone an MSC-like differentiation, and while they are heterogeneous, it is unknown which cell types are evident and how they facilitate the generation of the fat pad or immune privilege of the cells that allow for fat pad growth. Ongoing work will profile MEFs to determine if a population of cells primed for engrafting exists that is not present in the PDiPSCs. To facilitate engraftment, ongoing work is focused on using biomaterials or 3D constructs that can work synergistically with the designer adipocytes to help maintain them in the mouse (Yang *et al*., 2017), like Poly (ethylene glycol)-diacrylate (PEGDA) hydrogels or 3D woven scaffolds (Choi *et al*., 2021). Future steps for the designer gene knockout adipocytes will include creating more designer adipocyte knockout lines, including adipokines such as visfatin, resistin, and adipsin.

## Conclusions

The development of iPSC-derived adipocytes, or designer adipocytes, offers a unique opportunity to unravel the intricate roles of adipokines in health and disease progression. By leveraging CRISPR-Cas9 genome engineering in single cells that can be scaled rapidly, we can tightly control the cell identity to generate precise engineered tissue constructs. Through our research, we have demonstrated the potential of engineered adipose tissue as a novel drug delivery strategy and explored diverse designer adipocyte populations, including adipokine/gene knockout adipocytes, drug delivery adipocytes, and brown and white adipocytes. Despite the proof of concept demonstrated through the *in vivo* implantation studies in mice, there remains a need for a refined delivery approach, possibly through the integration of biomaterials or 3D scaffolds.

This work describes a potential framework for dissecting the functions of adipokines in disease progression, utilizing reprogrammed adipocytes as therapeutic depots and investigating the multifaceted roles of various adipose subtypes in disease and tissue therapeutics. The controlled system we propose, that spans from engineered from single cell isolates and implantable into fat-free mice for functional assessments, may address existing limitations in studying the downstream consequences of adipose signaling. The ultimate translational goal of this research is to employ the designer adipocytes developed in this study to meticulously dissect the involvement of adipokines in interorgan crosstalk, opening new avenues for understanding and targeting obesity-related diseases.

## Figures and Tables

**Table 1.** Criteria for chosen guide RNAs for Leptin KO. Score was computed as 100 % minus a weighted sum of off-target hit-scores in the target genome (full ranked list not shown). Single nucleotide polymorphism (SNP) NA indicates no common SNP found in the gRNA. Leptin KO iPSCs were then differentiated into PDiPSCs using the micromass procedure as described above.

Name	Score	Doench16	gRNA	SNP
YH665.m.Lep.sp7	96	0.604	CCCAATATGAATCCATCCAANGG	NA
YH666.m.Lep.sp9	86	0.624	GAATGATGGATGTGTGCACANGG	NA

Gene	Description	Primers
*R18s*	Housekeeper Gene	F: CGGCTACCACATCCAAGGAA R: GGGCCTCGAAAGAGTCCTGT
*Pparg*	Murine peroxisome proliferative activated receptor gamma	F: GCCCTGGAACTGCAGCTCAA R: GTGCTCTGTGACGATCTGCCT
*Apn*	Murine adiponectin	F: CCCTCCACCCAAGGGAACTT R: TCCAGGAGTGCCATCTCTGC
*Lep*	Murine leptin	F: GGGCTTCACCCCATTCTGAG R: AAGGCCAGCAGATGGAGGAG
*Ucp1*	Murine uncoupling protein 1	F: ACTGCCACACCTCCAGTCATT R: CTTTGCCTCACTCAGGATTGG
*Sox2*	Murine SRY-box 2	F: AGAAGGATAAGAGGAGCTGTG R: GGTAGGTATGGGAGGGTGATG
*Nanog*	Murine nanog homeobox	F: CGGACACTGGCTGAGAAGG R: GGTCTGCTGGAGGCTGAG
*Oct4*	Murine octamer-binding transcription factor 4	F: GAAAGCGGAGAAGGAGAGCA R: GGTCTCGTGGTTCTGTTGGC

## List of Abbreviations

iPSCs: induced pluripotent stem cells
WAT: white adipose tissue
BAT: brown adipose tissue
BeAT: beige adipose tissue
IL-1: Interleukin 1
IL-6: interleukin 6
TNF-α: tumor necrosis factor alpha
Ccl2: C-C motif chemokine ligand 2
LD: lipodystrophic
MEFs: mouse embryonic fibroblasts
WT: wildtype
Lep^−/−^: leptin knockout
Ccl2-Luc: Ccl2-Luciferase
PDiPSCs: pre-differentiated iPSCs
KO: knockout
ELISA: enzyme-linked immunosorbent assay
DI: deionized
IP: intraperitoneal injection
ROI: region of interest
ORO: Oil Red O
IL-1Ra: interleukin-1 receptor antagonist
SNP: single nucleotide polymorphism
PEGDA: Poly (ethylene glycol)-diacrylate
gRNAs: guide RNAs
PBS: phosphate-buffered saline
